# Interaction of *Bacteroides fragilis* and *Bacteroides thetaiotaomicron* with the kallikrein–kinin system

**DOI:** 10.1099/mic.0.046862-0

**Published:** 2011-07

**Authors:** Elizabeth C. Murphy, Matthias Mörgelin, Jakki C. Cooney, Inga-Maria Frick

**Affiliations:** 1 Department of Clinical Sciences, Lund, Division of Infection Medicine, Lund University, SE-22184 Lund, Sweden; 2 Department of Life Sciences and Materials and Surface Science Institute, University of Limerick, Limerick, Ireland

## Abstract

Many bacterial pathogens interfere with the contact system (kallikrein–kinin system) in human plasma. Activation of this system has two consequences: cleavage of high-molecular-mass kininogen (HK) resulting in release of the potent proinflammatory peptide bradykinin, and initiation of the intrinsic pathway of coagulation. In this study, two species of the Gram-negative anaerobic commensal organism *Bacteroides*, namely *Bacteroides fragilis* and *Bacteroides thetaiotaomicron*, were found to bind HK and fibrinogen, the major clotting protein, from human plasma as shown by immunoelectron microscopy and Western blot analysis. In addition, these *Bacteroides* species were capable of activating the contact system at its surface leading to a significant prolongation of the intrinsic coagulation time and also to the release of bradykinin. Members of the genus *Bacteroides* have been known to act as opportunistic pathogens outside the gut, with *B. fragilis* being the most common isolate from clinical infections, such as intra-abdominal abscesses and bacteraemia. The present results thus provide more insight into how *Bacteroides* species cause infection.

## INTRODUCTION

*Bacteroides* are obligately anaerobic, Gram-negative, pleomorphic rods that have a role in the human context as a member of the normal gut microbiota. *Bacteroides* *fragilis* is the type species of the genus. It is the most frequent species isolated from clinical infections and is considered to be the most virulent species, even though it is present in the gut at levels 100- to 1000-fold lower than other intestinal *Bacteroides* species. *Bacteroides* *thetaiotaomicron* is a prominent member of the distal intestinal microflora, comprising 6 % of all bacteria and 12 % of all *Bacteroides* in the human intestine. Apart from being a commensal bacteria in the gut, *Bacteroides* can also be opportunistic pathogens, mostly causing post-operative infections of the peritoneal cavity and bacteraemia (Wexler, 2007). Anaerobic infections involve many different bacteria, but *B. fragilis* is isolated from most infections. The mortality rate of infections caused by *B. fragilis*, which are treated appropriately, is 19 %. However, if the infection is left untreated, the mortality rate climbs to 60 % (Goldstein, 1996). *Bacteroides* are reported to have the most effective antibiotic resistance mechanisms of any anaerobic pathogen (Sóki *et al.*, 2006), with most members of the species being resistant to β-lactam agents (Nord & Olsson-Liljequist, 1981) and tetracycline (Kislak, 1972).

During establishment of a systemic infection such as bacteraemia, the invading organism has to contend with the full gambit of the host immune response. A component of this response, and one that is increasingly being recognized as an important aspect of the host immune defence, is contact activation of the intrinsic coagulation pathway. Activation of the contact system (kallikrein–kinin system) results in the initiation of procoagulative and proinflammatory cascades (Risberg *et al.*, 1991). This system encompasses three serine proteinases, factor XI (FXI), factor XII (FXII) and plasma prekallikrein (PK), and a non-enzymic co-factor, high-molecular-mass kininogen (HK). HK circulates in human plasma forming equimolar complexes with FXI and PK (Müller-Esterl, 1989). The system becomes activated upon exposure of FXII and HK to negatively charged surfaces. This causes the auto-activation of FXII to FXIIa and of PK to kallikrein. As a result, HK is cleaved by kallikrein releasing the nonapeptide bradykinin (Kaplan & Silverberg, 1987). FXI also becomes activated resulting in initiation of the intrinsic pathway of coagulation. Bradykinin is a proinflammatory peptide causing fever and pain, smooth-muscle contraction and increased vascular permeability by causing the production of prostaglandins and nitric oxide (Hall, 1992). Many bacterial pathogens, such as *Streptococcus pyogenes*, *Staphylococcus aureus* and *Salmonella*, can bind and assemble the components of the contact system leading to activation and bradykinin release (Frick *et al.*, 2007; Herwald *et al.*, 2003). In addition, recent work has shown that further processing of HK at the surface of bacterial pathogens generates antibacterial fragments from domain D3 of HK, including the 26 aa peptide NAT26 (Frick *et al.*, 2006). Thus, the contact system can be regarded as an important branch of innate immunity. Release of kinins at the site of bacterial infection has a dual role. On one hand, it is beneficial to the host as kinins aid in host defence to pathogens. They help in recruitment of defence cells such as neutrophils (Stuardo *et al.*, 2004) and monocytes (Barbasz *et al.*, 2008). On the other hand, release of bradykinin increases vascular permeability, which helps pathogens to colonize other tissues as well as allowing them access to more nutrients, due to increased infiltration of plasma into the site of infection (Karkowska-Kuleta *et al.*, 2009). Hence, contact system activation at the surface of bacterial cells can be seen as an important pathogenicity factor.

Despite the fact that *Bacteroides* are commonly isolated from clinical specimens, the pathogenic mechanisms of this bacterium are not well elucidated. Various virulence factors have been described, such as extracellular enzymes capable of degrading host molecules (Patrick, 2002) and binding to extracellular components such as fibronectin, collagen and vitronectin (Szöke *et al.*, 1996). The capsular polysaccharide complex was also thought to be a virulence factor (Krinos *et al.*, 2001). However, *Bacteroidetes* are commonly associated with opportunistic infection and also contain diverse polysaccharide loci, suggesting that the capsular polysaccharide may play more of a role in the prevention of bacteriophage infection in the gastrointestinal tract (Patrick *et al.*, 2010). A fibrinogen-binding protein, BF-FBP, was identified in *B. fragilis* and it was also shown that the bacterium possesses fibrinolytic activity (Houston *et al.*, 2010) and has an outer-membrane protein that interacts with components of the fibrinolytic system (Ferreira *et al.*, 2009). Recently, homologues of the streptococcal cysteine protease, SpeB, were identified in *B. fragilis* (Thornton *et al.*, 2010).

Given the ability of members of the genus *Bacteroides* to cause anaerobic infections, and the paucity of information on virulence mechanisms for these bacteria, we investigated whether the bacteria are capable of activation of the contact system. The results suggest that the ability of *Bacteroides* to cause infection may be aided by their ability to bind kininogen and fibrinogen from human plasma. The results also showed that the contact system could be activated on the surface of both *B. fragilis* and *B. thetaiotaomicron*. This resulted in a prolongation of the time taken to activate the intrinsic pathway of coagulation and a release of bradykinin from HK. During infection, all of these processes would allow the bacteria more access to nutrients due to serum seepage through an increase in vascular permeability. It would also give the bacteria greater ability to move outwards from the site of infection due to the binding of fibrinogen and contact factors, leading to a delay in coagulation.

## METHODS

### 

#### Bacteria, growth conditions and plasma sources.

The *B. fragilis* strain 638R was a kind gift from Dr Sheila Patrick, Queen's University, Belfast, Northern Ireland. The *B. thetaiotaomicron* strain VPI-5482 was purchased from the American Type Culture Collection. Bacteria were grown anaerobically overnight to stationary phase in BHI broth (Difco) supplemented with 5 µg haemin ml^−1^ (Sigma), 0.5 µg menadione ml^−1^ (Fluka) and 50 µg l-cysteine ml^−1^ (Sigma) at 37 °C for 18 h. Fresh frozen citrated plasma from healthy individuals was obtained from the blood bank at Lund University Hospital, Lund, Sweden, and kept frozen at −80 °C until use. Human kininogen-depleted plasma, human FXI-depleted plasma, human FXII-depleted plasma and human PK-depleted plasma were purchased from George King Bio-Medical.

#### Proteins, antibodies and reagents.

Human HK was purchased from Kordia and human fibrinogen was from Sigma. Anti-NAT26 antibodies were raised in rabbits as described previously (Frick *et al.*, 2006), rabbit anti-human fibrinogen was purchased from DakoCytomation, sheep anti-FXII from the Binding Site and goat anti-FXI from Affinity Biologicals. Sheep anti-human HK (AS88) and rabbit anti-PK were a kind gift from Heiko Herwald, Lund University, Sweden. Horseradish peroxidase (HRP)-conjugated goat anti-rabbit IgG was purchased from Pierce, HRP-conjugated donkey anti-sheep IgG from MP Biomedicals and HRP-conjugated Protein A from Sigma. The FXII/plasma kallikrein inhibitor H-D-Pro-Phe-Arg-chloromethylketone (CMK) peptide was purchased from Bachem Feinchemikalien.

#### Plasma absorption assay.

An overnight culture of bacteria grown to stationary phase was washed twice and resuspended in PBS (116 mM NaCl, 1.7 mM KH_2_PO_4_, 4.9 mM Na_2_HPO_4_ . 2H_2_O). One millilitre of a 2×10^9^ bacteria ml^−1^ suspension was incubated with 1 ml citrated human plasma for 1 h at 37 °C. As a control, bacteria were also incubated with PBS. The bacterial cells were recovered and washed with PBS four times to remove any unbound plasma proteins. Bound proteins (proteins bound to the surface of the bacteria and not removed by washing) were eluted with 0.1 M glycine/HCl, pH 2.0, and 1 M Tris (unbuffered) was added to the eluted proteins to raise the pH to 7.5. Eluted proteins were analysed by SDS-PAGE and Western blotting.

#### Contact factor and fibrinogen binding studies.

An overnight culture of bacteria grown to stationary phase was washed twice and resuspended in PBS. One hundred microlitres of a 2×10^9^ bacteria ml^−1^ suspension was incubated with 100 µl PBS, 80 µg HK ml^−1^ and FXI/FXII/PK, respectively, for 1 h at 37 °C. The contact factors were used at human plasma concentrations (5 µg FXI ml^−1^, 30 µg FXII ml^−1^ and 40 µg PK ml^−1^). As a control, bacteria were also incubated with PBS only. For the fibrinogen binding studies, 100 µl of a 2×10^9^ bacteria ml^−1^ suspension was incubated with 100 µl PBS and 300 µg fibrinogen ml^−1^ for 1 h at 37 °C. The bacterial cells were recovered and washed with PBS four times to remove any unbound plasma proteins. Bound proteins (proteins bound to the surface of the bacteria and not removed by washing) were eluted with 0.1 M glycine/HCl, pH 2.0, and 1 M Tris (unbuffered) was added to the eluted proteins to raise the pH to 7.5. Eluted proteins were precipitated with 5 % trichloroacetic acid (TCA) for 30 min on ice followed by centrifugation at 15 000 ***g*** (4 °C for 20 min). Precipitated material was dissolved in SDS sample buffer and analysed by SDS-PAGE and Western blotting.

#### Enzyme digestions and affinity chromatography.

Bacteria were cultivated overnight to stationary phase and digestions of bacteria with papain, pepsin and trypsin were performed as described by Björck (1988). For mutanolysin digestion, the bacteria were washed and resuspended in 0.01 M KH_2_PO_4_, pH 6.8, to 2×10^10^ bacteria ml^−1^. To 1 ml bacterial suspension, 10 U mutanolysin (Sigma) and 2 µg DNase were added, and the mixture was incubated at 37 °C for 2 h. To terminate the reaction, pH was adjusted to 7.5 with 7.5 % (w/v) NaHCO_3_. Bacteria were pelleted by centrifugation at 4000 ***g*** at 4 °C for 15 min and the remaining supernatant was subjected to affinity chromatography by using fibrinogen coupled to cyanogen bromide (CNBr)-activated Sepharose 4B (GE-Healthcare) as described by the manufacturer. Following extensive washing with PBS, bound proteins were eluted with 0.1 M glycine/HCl, pH 2.0, and the pH was raised to approximately 7.4 with 1 M Tris (unbuffered). Fractions were subjected to SDS-PAGE and Western blot analysis. As a control, the mutanolysin-released surface proteins were also subjected to a non-conjugated CNBr-activated Sepharose 4B column to ensure no non-specific interactions were taking place with the resin.

#### SDS-PAGE and Western blot analysis.

SDS-PAGE was performed as described by Neville (1971). Samples were prepared for loading by boiling in sample buffer containing 2 % SDS and 5 % β-mercaptoethanol for 5 min, and 12 and 15 % gels were used to resolve plasma proteins eluted from the bacterial surface. Separated proteins were visualized by Coomassie blue staining. For Western blot analysis, proteins were electrophoretically transferred to a PVDF membrane (Amersham Biosciences). Subsequently, the membrane was blocked in a PBS-Tween [PBS containing 0.1 % Tween 20 (PBS-T)] solution containing 5 % (w/v) skimmed milk powder at 37 °C for 30 min. Membranes were washed three times with PBS-T for 5 min followed by incubation with primary antibodies (rabbit anti-NAT26, 1 : 100 dilution; rabbit anti-human fibrinogen, 1 : 1000 dilution; sheep anti-human FXII, 1 : 1000 dilution; goat anti-human FXI, 1 : 1000 dilution; rabbit anti-human PK, 1 : 1000 dilution) in blocking buffer at 37 °C for 30 min. Membranes were washed three times with PBS-T for 5 min followed by incubation with HRP-conjugated secondary antibodies (protein A, 1 : 5000 dilution; goat anti-rabbit IgG, 1 : 5000 dilution; or donkey anti-sheep IgG, 1 : 5000 dilution) in blocking buffer at 37 °C for 30 min. Following a repeat of the wash steps, bound antibodies were detected by chemiluminesence as described by Nesbitt & Horton (1992).

#### Clotting assays.

An overnight culture of bacteria grown to stationary phase was washed with 13 mM sodium citrate solution and made to a concentration of 2×10^10^ bacteria ml^−1^ by using the same buffer. One hundred microlitres of human plasma was incubated with 30 µl of the bacterial suspension for 1 h at 37 °C. The bacteria were recovered by centrifugation at 13 000 ***g*** at room temperature for 1 min and the supernatant was used in the clotting assays. For the intrinsic pathway of coagulation [activated partial thromboplastin time (aPTT) assay], 100 µl DAPTTIN (double activated aPTT) (Technoclone) was incubated with 100 µl of supernatant for 200 s at 37 °C in a coagulometer (Amelung). Then, 100 µl of 25 mM CaCl_2_ was added and the time taken to form a clot was measured. For the extrinsic pathway of coagulation [prothrombin (PT) time assay], 100 µl TriniCLOT PT Excel (Kordia) was added to 100 µl supernatant in a coagulometer at 37 °C, and the time taken to form a clot was measured. For the common pathway of coagulation [thrombin (TCT) time], 100 µl thrombin (Hyphen Biomed) was added to 100 µl supernatant in a coagulometer at 37 °C, and the time taken to form a clot was measured.

#### Assay for FXIIa, FXIa and activated PK.

An overnight culture of bacteria grown to stationary phase was washed with 13 mM sodium citrate and made to a concentration of 2×10^10^ cells ml^−1^ by using the same buffer. One hundred microlitres of human plasma was incubated with 30 µl of the bacterial suspension for 30 min at 37 °C. Plasmas deficient in FXII, FXI and PK were used as controls. Bacteria were recovered by centrifugation at 13 000 ***g*** at room temperature for 1 min and the supernatant was discarded. The cell pellet was washed once with 200 µl 13 mM sodium citrate solution to remove unbound plasma proteins and subsequently resuspended in 100 µl of S-2302 chromogenic substrate (Chromogenix). The mixture was incubated at room temperature for 30 min. The bacterial cells were recovered as before and 100 µl of the supernatant was transferred to an ELISA plate. The absorbance of the substrate was measured at 405 nm. S-2302 is a fluorescent substrate for FXIIa, FXIa and activated PK in plasma. An increase in absorbance is indicative of deposition of activated plasma components at the cell. Following resuspension of bacterial cells in the chromogenic substrate, a time-dependent recording of protease activity was also performed directly in the bacterial suspensions.

#### Detection of bradykinin by ELISA.

Bacterial samples for ELISA were prepared by preincubating 100 µl of a 2×10^10^ bacteria ml^−1^ suspension, from a culture grown overnight to stationary phase, with 100 µl human plasma at room temperature for 15 min. Following incubation, the bacteria were recovered as above and the supernatant was discarded. Pellets were resuspended in 50 mM Tris-HCl, pH 7.5, containing 50 µM ZnCl_2_ and allowed to stand at room temperature for 15 min. Following incubation, the cells were recovered by centrifugation as described above, and the supernatant was transferred to a fresh tube and 10 µl TCA solution (provided with the ELISA kit) was added. The resulting precipitate was removed by centrifugation at 14 000 ***g*** for 2.5 min at room temperature. The supernatant (50 µl) was transferred to a fresh tube containing 50 µl Buffer B (from Markit-M Bradykinin kit). Samples were stored at −20 °C for further analysis. The bradykinin ELISA was carried out by using the Markit-M Bradykinin kit according to the manufacturer's instructions. Absorbance was measured at 405 nm, and the concentration of bradykinin in the samples was calculated by using a standard curve.

#### MS and N-terminal amino acid sequencing.

Protein bands on SDS-PAGE were excised and subjected to in-gel trypsination and MS/MS analysis. This was performed at the SCIBLU-Swegene Centre for Integrative Biology at Lund University, Sweden. The residues of the individual peptide sequence interpretations were used for a search of the *B. fragilis* 638R sequence by using Artemis sequence visualization software (Rutherford *et al.*, 2000). N-terminal amino acid sequencing was performed on an ABI Procise 494 sequencer at Alphalyse.

#### Thin-sectioning and transmission electron microscopy.

An overnight culture of bacteria grown to stationary phase was washed with 13 mM sodium citrate and adjusted to 2×10^10^ bacteria ml^−1^ by using the same buffer. Thirty microlitres of the bacterial suspension was incubated with 100 µl citrated human plasma or buffer for 1 h at 37 °C. Following incubation, the bacteria were recovered by centrifugation at 3000 ***g*** and fixed for 1 h at room temperature and then overnight at 4 °C in 2.5 % (v/v) glutaraldehyde in 0.15 M sodium cacodylate, pH 7.4 (cacodylate buffer). Samples were washed with cacodylate buffer, dehydrated in a graded series of ethanol solutions and then embedded in Epon 812 by using acetone as an intermediate solvent. Specimens were sectioned with a diamond knife into 50-nm-thick ultrathin sections on an LKB ultramicrotome and mounted on gold grids. For immunostaining, grids were floated on top of drops of immune reagents displayed on a sheet of Parafilm. Free aldehyde groups were blocked with 50 mM glycine in PBS, followed by incubation with 5 % (v/v) goat serum in incubation buffer [0.2 % BSA-c in PBS (acetylated bovine serum albumin from Aurion), pH 7.6] for 15 min. This blocking procedure was followed by overnight incubation at 4 °C with rabbit anti-human fibrinogen (diluted 1 : 10 in incubation buffer), rabbit anti-NAT26 (diluted 1 : 10 in incubation buffer) or sheep anti-human HK AS88 (diluted 1 : 10 in incubation buffer), respectively. After washing the grids in a large volume (200 ml) of incubation buffer, floating on drops containing gold-conjugated reagent (goat anti-rabbit IgG coupled to 10-nm gold particles at 1 µg ml^−1^ in incubation buffer, or donkey anti-sheep IgG coupled to 5-nm gold particles at 1 µg ml^−1^ in incubation buffer) was performed for 2 h at 4 °C. After further washes in a large volume of incubation buffer, the sections were post-fixed in 2 % glutaraldehyde. Finally, sections were washed with distilled water, post-stained with 2 % uranyl acetate and lead citrate, and examined with a JEOL JEM 1230 electron microscope operated at 80 kV accelerating voltage. Images were recorded with a Gatan Multiscan 791 charge-coupled device camera.

## RESULTS

### HK binds to the surface of *B. fragilis* and *B. thetaiotaomicron*

**Fig. 1.**
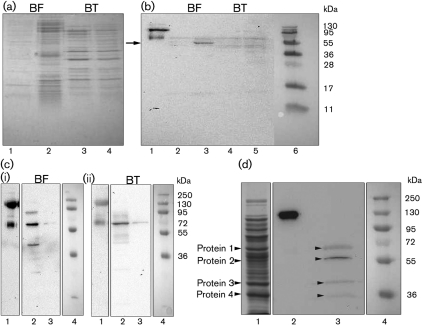
*Bacteroides* bind HK. (a) *B. fragilis* and *B. thetaiotaomicron* were incubated with human plasma or PBS for 1 h at 37 °C. Bacteria were washed, and bound plasma proteins were eluted at low pH and subjected to SDS-PAGE. The gel was stained with Coomassie blue. Lanes: 1, *B. fragilis*+PBS; 2, *B. fragilis*+plasma; 3, *B. thetaiotaomicron*+PBS; 4, *B. thetaiotaomicron*+plasma. (b) Western blot analysis of the samples in (a) using antibodies against the NAT26 peptide derived from domain D3 of HK as probe. Lanes: 1, native HK; 2, *B. fragilis*+PBS; 3, *B. fragilis*+plasma; 4, *B. thetaiotaomicron*+PBS; 5, *B. thetaiotaomicron*+plasma; 6, prestained protein marker. The arrow points to the HK fragment bound by *B. fragilis*. (c) Binding of HK to the surface of *B. fragilis* (i) and *B. thetaiotaomicron* (ii). Lanes: 1, native HK; 2, *B. fragilis*/*B. thetaiotaomicron*+HK; 3, *B. fragilis*/*B. thetaiotaomicron*+PBS; 4, prestained protein marker. (d) Proteins were released from the surface of *B. fragilis* by mutanolysin treatment, precipitated with TCA and subjected to SDS-PAGE. Separated proteins were transferred to a PVDF membrane, which was incubated with HK (20 µg ml^−1^) followed by anti-NAT26 antibodies. Four protein fragments interacting with HK were identified (indicated by numbers). Lanes: 1, SDS profile of mutanolysin extract; 2, native HK; 3, surface proteins of *B. fragilis* binding to HK; 4, prestained protein marker.

To investigate if *Bacteroides* can take up HK, the precursor of bradykinin, from plasma, *B. fragilis* and *B. thetaiotaomicron* were incubated with human plasma for 1 h at 37 °C. Bacteria incubated with PBS were used as a control. Following incubation, the bacterial cells were washed, and bound proteins were eluted with a low-pH buffer and analysed by SDS-PAGE (Fig. 1a). *B. fragilis* appears to bind a broader range of plasma proteins than *B. thetaiotaomicron*. Furthermore, by comparison with the control it can be seen that most of the proteins in the *B. thetaiotaomicron* sample appear to be of bacterial origin, presumably outer-membrane proteins released under the low-pH conditions (Fig. 1a). Western blot analysis with an anti-NAT26 antibody, which reacts with the D3 domain of the heavy chain of HK, demonstrated that this protein was bound to the surface of *B. fragilis* (Fig. 1b, lane 3). When the contact system is activated, PK cleaves native HK (120 kDa) into a heavy chain of ~65 kDa and a light chain of ~55 kDa. HK eluted from *B. fragilis* was cleaved into a smaller fragment (the size of the immunoreactive band was only ~55 kDa, Fig. 1b). This fragment is most likely generated by further processing of the HK heavy chain.

Next, *B. fragilis* and *B. thetaiotaomicron* were incubated with purified intact HK at a concentration corresponding to that of human plasma (80 µg ml^−1^) for 1 h at 37 °C. Bacteria were also incubated with PBS only as a negative control. In contrast to the plasma absorption experiment, both *Bacteroides* species bound purified HK, as demonstrated by Western blot analysis using antibodies against NAT26 (Fig. 1c). Furthermore, bound HK was degraded (Fig. 1c), suggesting that, in the absence of activated PK, HK is probably cleaved by a protease expressed by the bacteria. Such activity has been described for proteases from various bacterial species (Frick *et al.*, 2007; Imamura *et al.*, 2004).

To release the HK binding protein from the bacterial cell surface, *B. fragilis* was treated with pepsin, papain, trypsin or mutanolysin as described by Björck (1988). The proteins released from *B. fragilis* were analysed by SDS-PAGE and Western blotting, and although mutanolysin has mainly been used for digestion of Gram-positive bacterial cell walls (Yokogawa *et al.*, 1974), this enzyme was the most efficient in releasing membrane proteins of *B. fragilis*. The material extracted with mutanolysin (Fig. 1d, lane 1) contained four protein fragments, which reacted with HK (Fig. 1d, lane 3). This was demonstrated through incubation of the blot with HK followed by detection of HK binding through the anti-NAT26 antibody. These four proteins were subjected to MS/MS analysis (Fig. 1d). Protein nos 1 and 4 were identified as a chaperone GroEL and a malate dehydrogenase, respectively. Protein no. 3 was identified as a 41 kDa hypothetical protein and protein no. 2 as a 60.5 kDa hypothetical protein with a SusD/RagB domain. A blast analysis of protein 2 showed that it had high homology to putative outer-membrane proteins and proteins involved in nutrient binding with a SusD/RagB domain. Due to its size, location and strongest reactivity in the Western blot, protein 2 (BF638R1721) is suggested to be a putative HK binding protein at the surface of *B. fragilis.*

### *B. fragilis* and *B. thetaiotaomicron* bind and activate contact factors on their surface

**Fig. 2.**
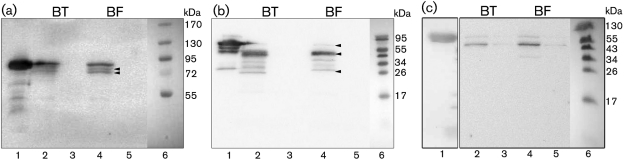
*B. fragilis* and *B. thetaiotaomicron* activate the contact system. (a) Binding of FXI to the surface of *Bacteroides*. Lanes: 1, native FXI; 2, *B. thetaiotaomicron*+FXI; 3, *B. thetaiotaomicron*+PBS; 4, *B. fragilis*+FXI; 5, *B. fragilis*+PBS; 6, prestained protein marker. The arrowheads point to activated conformations of FXI. (b) Binding of FXII to the surface of *Bacteroides*. Lanes: 1, native FXII; 2, *B. thetaiotaomicron*+FXII; 3, *B. thetaiotaomicron*+PBS; 4, *B. fragilis*+FXII; 5, *B. fragilis*+PBS; 6, prestained protein marker. The arrowheads point to intact FXII, HCFXII and LCFXII, respectively. (c) Binding of PK to the surface of *Bacteroides*. Lanes: 1, native PK; 2, *B. thetaiotaomicron*+PK; 3, *B. thetaiotaomicron*+PBS; 4, *B. fragilis*+PK; 5, *B. fragilis*+PBS; 6, prestained protein marker.

To examine further the interaction of contact factors with the bacterial surface of *Bacteroides*, *B. fragilis* and *B. thetaiotaomicron* were incubated with HK together with either FXI, FXII or PK in a PBS solution for 1 h at 37 °C. All contact factors were used at concentrations corresponding to those in human plasma. As a control, the bacteria were incubated with PBS only. Proteins bound to the bacteria were eluted at low pH, followed by Western blot analysis and detection of bound protein with antibodies. Co-incubation with FXI showed that both bacteria were capable of binding and activating FXI on their surface (Fig. 2a). There are no bands in the PBS control fraction; therefore, the bands in lanes 2 and 4 are associated with FXI binding. Activation of FXI proceeds through cleavage of the Arg369–Ile370 peptide bonds on both subunits of the dimer. This proceeds through an intermediate with one activated subunit, referred to as ½-FXIa (Emsley *et al.*, 2010). The presence of three bands on the blot suggests the existence of inactive, intermediate and fully activated FXI on the bacterial surface, as different protein conformations migrate at slightly different rates (Emsley *et al.*, 2010). Co-incubation of both bacterial species with FXII produced a similar outcome (Fig. 2b). The two immunoreactive bands of approximately 52 and 28 kDa correspond to the heavy chain (HCFXII) and light chain (LCFXII) components of FXII upon cleavage (Harris *et al.*, 2005). A very faint band is observed at 80 kDa, the molecular mass of intact FXII. Therefore, the majority of FXII has been activated on the bacterial surface. Investigation of the binding of PK to the surface of *B. fragilis* and *B. thetaiotaomicron* revealed an immunoreactive fragment of approximately 55 kDa, suggesting binding and activation of PK by both bacteria (Fig. 2c).

**Fig. 3.**
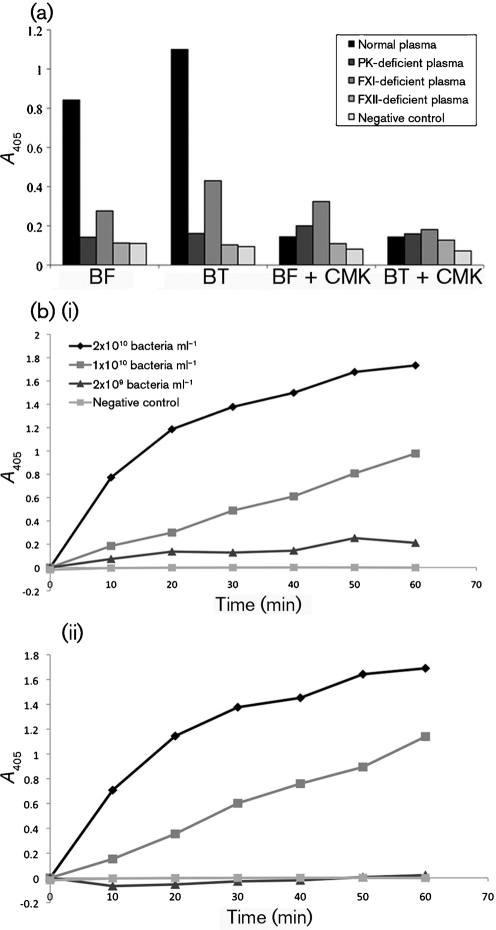
Determination of contact system activation by *Bacteroides* by using a chromogenic substrate. (a) Examination of contact system activation by *B. fragilis* and *B. thetaiotaomicron* in normal plasma and plasma deficient in PK, FXI or FXII, and a negative control (13 mM sodium citrate buffer) by using the S-2302 chromogenic substrate. The bacteria were also incubated with an inhibitor of FXII and PK activation, CMK. Experiments were repeated three times, and a representative experiment is shown. (b) Examination of contact system activation by *Bacteroides*, at the indicated bacterial concentrations, by using the S-2302 chromogenic substrate in a time-dependent manner: (i) *B. fragilis* and (ii) *B. thetaiotaomicron*. The experiment was performed once.

The chromogenic substrate S-2302 can be used to analyse the presence of active PK, FXIIa and FXIa by monitoring fluorescence as a result of hydrolysis of S-2302. Therefore, the interaction of the contact factors with the surface of *Bacteroides* was further investigated by incubating the bacteria with human plasma and then testing for the presence of PK, FXIIa and FXIa by hydrolysis of S-2302. For *B. fragilis* and *B. thetaiotaomicron* cells incubated in normal plasma, there was an increased fluorescence compared with controls, indicating that the contact system had been activated (Fig. 3a). In additional experiments, bacteria were incubated with plasma deficient in either PK, FXI or FXII and with 13 mM sodium citrate solution (negative control). The negative control gave a mean absorbance that was only 11 % of maximal activation. This was comparable with that of plasma deficient in FXII. The plasma deficient in PK gave an absorbance that amounted to 16 % of maximal activation, probably as a result of activated FXI and FXII present in the kallikrein-depleted plasma. Finally, the plasma deficient in FXI gave an absorbance corresponding to 36 % of the maximal activation caused by bacteria incubated in normal plasma. This activity can be accounted for by the presence of activated FXII and kallikrein in the plasma. In the presence of H-D-Pro-Phe-Arg-CMK, an inhibitor of FXII and PK activation, contact activation was efficiently blocked by both bacteria in normal plasma (Fig. 3a). In addition, time-dependent recording of the protease activity clearly demonstrated the presence of active contact factors on the bacterial surface (Fig. 3b).

Overall, these results strongly suggest that *B. fragilis* and *B. thetaiotaomicron* are capable of activating the human contact system through recruitment and activation of contact factors on the bacterial surface.

### *B. fragilis* interacts with fibrinogen via an outer-membrane protein

**Fig. 4.**
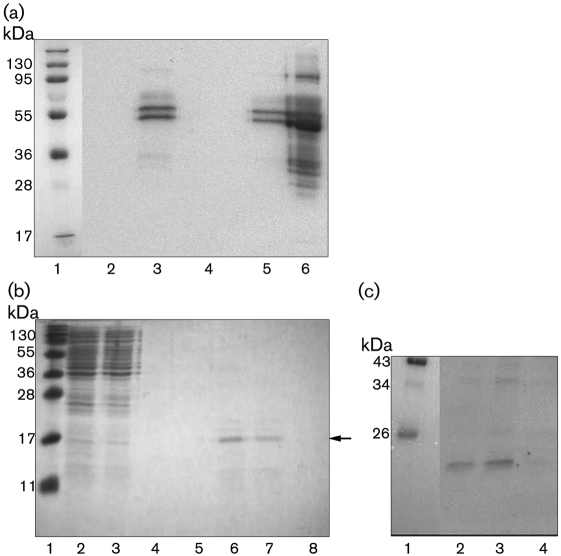
*Bacteroides* bind fibrinogen. (a) *B. fragilis* and *B. thetaiotaomicron* were incubated with fibrinogen (300 µg ml^−1^) or PBS. Bound protein was eluted, analysed by Western blotting and probed with anti-fibrinogen antibodies. Lanes: 1, prestained protein marker; 2, *B. fragilis*+PBS; 3, *B. fragilis*+fibrinogen; 4, *B. thetaiotaomicron*+PBS; 5, *B. thetaiotaomicron*+fibrinogen; 6, human fibrinogen. (b) Coomassie blue-stained gel showing enrichment of an 18 kDa protein that binds to fibrinogen coupled to Sepharose. Lanes: 1, prestained protein marker; 2, proteins released from the surface of *B. fragilis* by mutanolysin; 3, run-through from the column; 4–8, protein eluted in fractions from the column. The band indicated with an arrow was subjected to N-terminal amino acid sequencing. (c) Purified proteins from (b) were transferred to a PVDF membrane, incubated with a 20 µg fibrinogen ml^−1^ solution and probed with an anti-fibrinogen antibody. Lanes: 1, prestained protein marker; 2–4, protein eluted in fractions from the fibrinogen–Sepharose column.

It was recently shown that *B. fragilis* binds and degrades purified human fibrinogen, a major clotting protein of plasma (Houston *et al.*, 2010). Thus, the plasma proteins bound by both *B. fragilis* and *B. thetaiotamicron* (Fig. 1a) were also analysed for fibrinogen binding by Western blotting by using an anti-fibrinogen antibody as a probe. However, a very low level of reaction was obtained (data not shown). Therefore, *B. fragilis* and *B. thetaiotaomicron* were incubated with human fibrinogen followed by Western blot analysis with antibodies against fibrinogen. This blot showed a definite interaction between both *B. fragilis* and *B. thetaiotaomicron* with fibrinogen (Fig. 4a). The outer-membrane proteins released from *B. fragilis* by mutanolysin were then tested in a Western blot for a possible interaction with fibrinogen. Following incubation with human fibrinogen, bound protein was detected with an anti-fibrinogen antibody. However, due to weak binding, this blot did not give a clear indication of which outer-membrane proteins from *B. fragilis* were capable of binding fibrinogen (data not shown). Therefore, the proteins released by mutanolysin were applied to affinity chromatography by using fibrinogen-coupled Sepharose. A protein of approximately 18 kDa was eluted from the fibrinogen–Sepharose (Fig. 4b, lanes 6 and 7) and subjected to N-terminal amino acid sequencing. The same procedure was carried out on a non-conjugated CNBr-activated Sepharose column and no proteins were purified (data not shown), signifying a specific interaction of the cell surface protein with fibrinogen. Furthermore, the affinity purified fractions were subjected to Western blot analysis to confirm fibrinogen interaction. The blot was incubated with a fibrinogen solution and bound protein was detected with anti-fibrinogen antibodies (Fig. 4c). The immunoreactive band at approximately 18 kDa shows that the purified protein does, in fact, bind fibrinogen. A search of the *B. fragilis* 638R genome with the obtained N-terminal sequence (LMGEA) identified it as being internal to a hypothetical protein of 57.7 kDa (BF638R0397). The size of the protein fragment starting at LMGEA is 21 kDa. Although this fragment is larger than the detected 18 kDa protein, it is possible that both N- and C-terminal processing of the 57.7 kDa precursor has occurred. One other hit for the amino acid sequence LMGEA was identified in the translated protein repertoire from *B. fragilis* 638R. This sequence was within a protein (BF638R1117) and very close to the C terminus and would result in a 12.6 kDa protein, which is too small to be the protein eluted from the fibrinogen-coupled Sepharose column. A blast (Altschul *et al.*, 1990) analysis of the amino acid sequence of the 57.7 kDa protein showed that it contained a SusD/RagB domain and that it had high homology to outer-membrane proteins and proteins with a SusD/RagB domain. There was 18 % identity and 29 % similarity to the recently published fibrinogen-binding protein in *B. fragilis*, BF-FBP (Houston *et al.*, 2010), suggesting that *Bacteroides* absorbs fibrinogen to its surface via several proteins. This result suggests that *B. fragilis* has two surface proteins capable of binding human fibrinogen. Alignment of this protein to the potential HK binding protein identified above was carried out by using the ‘blast 2 sequences’ program (Altschul *et al.*, 1990). This analysis revealed that the proteins are 25 % identical and 40 % similar. Therefore, there is a significant level of similarity between these two proteins.

### *B. fragilis* and *B. thetaiotaomicron* cause prolongation of the intrinsic pathway of clotting in human blood

Due to the ability of *Bacteroides* to bind fibrinogen and to investigate further the bacterial interaction with the contact system, the effect of the bacteria on different pathways of coagulation was examined. All three pathways of clotting were tested (assays used to test them in parentheses): the intrinsic pathway (aPTT), the extrinsic pathway (PT) and the common pathway (TCT). The extrinsic pathway of coagulation is initiated in response to trauma, where tissue factor becomes exposed and binds to the activated form of factor VII (Dahlbäck, 2000). The intrinsic pathway of coagulation is initiated by activation of the contact system, where FXII activates FXI in its complex with HK. Initiation of this pathway is not associated with trauma-initiated coagulation and the mode of activation on cellular surfaces has not yet been fully elucidated (Dahlbäck, 2000; Oehmcke & Herwald, 2010). However, once activated, the contact system is involved in regulation of haemostatic and inflammatory processes (Oehmcke & Herwald, 2010).

**Fig. 5.**
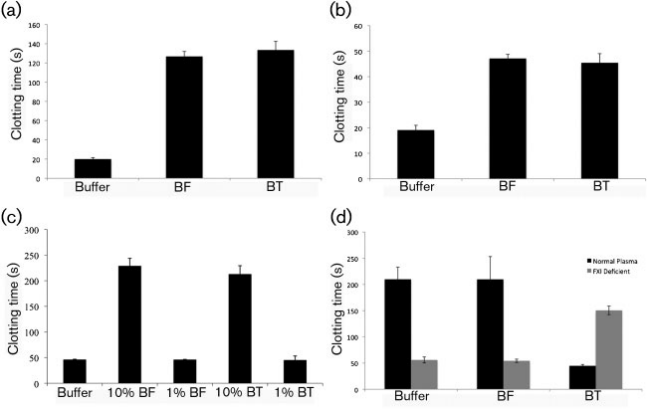
Effect of *Bacteroides* on the intrinsic, extrinsic and common pathways of coagulation. (a–c) *B. fragilis* (BF) and *B. thetaiotaomicron* (BT) were incubated with human citrated plasma for 1 h at 37 °C. Bacteria were removed and the resulting plasma supernatants were analysed by clot formation tests. For the negative control, bacteria were substituted with 13 mM sodium citrate solution. Data are means±sd from three experiments. (a) PT clot formation test, (b) TCT clot formation test and (c) aPTT clot formation test. (d) *B. fragilis* and *B. thetaiotaomicron* were incubated with human citrated plasma for 1 h at 37 °C. Bacteria were harvested and the resulting plasma supernatants were analysed by the aPTT clot formation test. The resulting bacterial pellets were resuspended in 100 µl FXI-deficient plasma, and clot formation was also analysed. For the negative control, bacteria were substituted with 13 mM sodium citrate buffer. Data are means±sd from three experiments.

*B. fragilis* and *B. thetaiotaomicron* caused a significant delay in the clotting times for all three pathways. The TriniCLOT reagent used in the PT assay is sensitive to deficiencies in factors VII and X. This prolongation of the extrinsic pathway of coagulation times suggests that *Bacteroides* may be binding these clotting factors to their surface, leading to a depletion in plasma (Fig. 5a). The TCT for the common pathway of coagulation was also significantly delayed (Fig. 5b). This result suggests a depletion of fibrinogen in plasma, which agrees with the finding that both *B. fragilis* and *B. thetaiotaomicron* interact with fibrinogen and also with the recent identification of a fibrinogen-binding protein on the surface of *B. fragilis* (Houston *et al.*, 2010). In addition, both bacterial species had a significant effect on the intrinsic pathway (Fig. 5c), causing a prolongation of clotting times in the aPTT assay compared with the negative control (13 mM sodium acetate buffer), with *B. fragilis* (228 s mean clotting time) being slightly more effective than *B. thetaiotaomicron* (212 s mean clotting time). As LPS of Gram-negative bacteria has been shown to activate the contact system (Kalter *et al.*, 1983), the aPTT assay was also conducted by adding polymyxin B to neutralize the effect of LPS (Cardoso *et al.*, 2007). In the presence of polymyxin B at neutralizing concentrations, both *Bacteroides* species still significantly delayed the clotting time (data not shown), demonstrating that other components on the bacterial surface are responsible for binding and assembly of the contact factors.

A further clotting experiment was carried out by using the aPTT assay to investigate clotting times of bacteria pre-incubated in normal plasma for 1 h at 37 °C, harvested and resuspended in FXI-deficient plasma. The control, which consisted of FXI-deficient plasma and sodium citrate buffer, had a mean clotting time of 151 s. This clotting time is significantly longer than in normal plasma (45 s) due to the absence of FXI, which is necessary to initiate the intrinsic pathway of coagulation (Fig. 5d). However, bacterial pellets that were resuspended in FXI-deficient plasma had a mean clotting time of 56 s for *B. fragilis* and 54 s for *B. thetaiotaomicron* in the aPTT assay (Fig. 5d). This result signifies binding and activation of FXI on the surface of both bacteria during incubation in normal plasma. Upon resuspension in FXI-deficient plasma, the FXIa bound to the surface of the bacteria initiates the intrinsic pathway of coagulation, and clotting times are almost that of the normal plasma control (45 s).

Prolongation of clot formation is a sign of a deficiency of coagulation factors such as contact-phase proteins and fibrinogen (Ponjee *et al.*, 1991). Therefore, it would appear that both bacteria are capable of activating the contact system.

### *B. fragilis* and *B. thetaiotaomicron* induce release of bradykinin from HK in human plasma

**Fig. 6.**
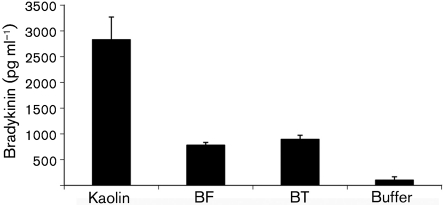
Bradykinin is released by *B. fragilis* (BF) and *B. thetaiotaomicron* (BT)*.* Bacteria were incubated with human plasma (final concentration 50 %) as described in Methods. Kaolin was used as a positive control and buffer was used as a negative control. Released bradykinin was determined by ELISA. Data are means±sd from three experiments.

As a consequence of contact activation, bradykinin is released from HK through cleavage by PK. Bradykinin is a potent proinflammatory peptide and its release causes many inflammatory responses. These include increased microvascular permeability, smooth-muscle contraction, fever and pain (Bhoola *et al.*, 1992; Hall, 1992). *B. fragilis* and *B. thetaiotaomicron* were incubated in human plasma and bradykinin release was measured by using the MARKIT-M bradykinin ELISA kit. Kaolin was used as a positive control as it has a negatively charged surface and causes massive activation of the contact system. In this system, kaolin activation resulted in release of 2830 pg bradykinin ml^−1^, while the negative control, 50 mM Tris-HCl, pH 7.5, containing 50 µM ZnCl_2_, released 103 pg ml^−1^ (Fig. 6). Although values were lower than the positive control, both bacterial species caused a significant release of bradykinin. *B. thetaiotaomicron* caused a release of 895 pg ml^−1^ and appears to be slightly more effective than *B. fragilis*, which caused a release of 781 pg bradykinin ml^−1^ (Fig. 6). In the presence of the FXII and PK inhibitor H-D-Pro-Phe-Arg-CMK, bradykinin release by the bacteria was completely inhibited (data not shown). Hence, it would appear that bradykinin release is occurring on the surface of the bacteria as a result of contact activation.

Further processing of HK at the surface of bacterial pathogens has been shown to generate antibacterial fragments of HK, mainly involving the peptide sequence NAT26 (Frick *et al.*, 2006). However, no such fragments were observed when either of the *Bacteroides* species in this study was incubated in human plasma (Fig. 1b).

### Analysis of fibrinogen and HK binding to the surface of *B. fragilis* and *B. thetaiotaomicron* by electron microscopy

**Fig. 7.**
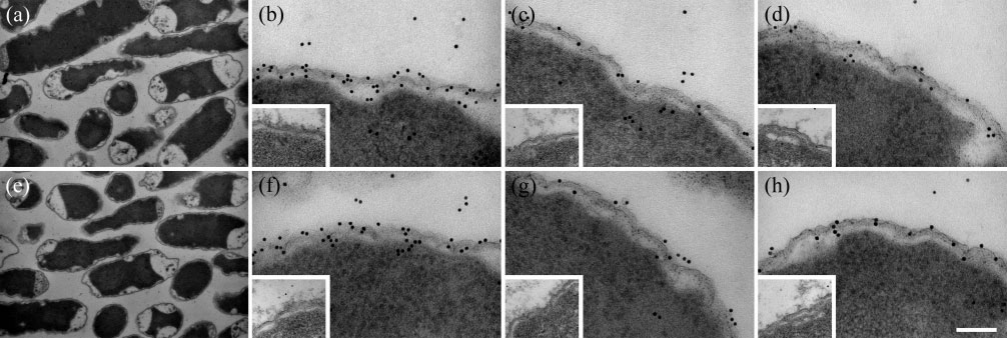
Transmission electron micrographs of *B. fragilis* and *B. thetaiotaomicron* incubated in human plasma. Following incubation, the bacteria were prepared for ultrathin sectioning/immunoelectron microscopy. Sections were incubated with primary antibodies to human fibrinogen, HK AS88 or NAT26, followed by secondary antibodies (1 µg ml^−1^) labelled with colloidal gold. (a) *B. fragilis* incubated in plasma for 1 h, (b) *B. fragilis* probed with rabbit anti-human fibrinogen antibody, (c) *B. fragilis* probed with anti-HK AS88, (d) *B. fragilis* probed with anti-NAT26, (e) *B. thetaiotaomicron* incubated in plasma for 1 h, (f) *B. thetaiotaomicron* probed with rabbit anti-human fibrinogen antibody, (g) *B. thetaiotaomicron* probed with anti-HK AS88; and (h) *B. thetaiotaomicron* probed with anti-NAT26. The inset images represent the negative control which was *Bacteroides* incubated with sodium citrate buffer only instead of human plasma. Bars, 1 µm for (a) and (e), 100 nm for (b)–(d) and (f)–(h).

Binding of HK and fibrinogen to the bacteria was examined visually by using immunoelectron microscopy. Samples were prepared by preincubating bacteria with human plasma for 1 h at 37 °C followed by thin sectioning of the bacteria. The apparent shrinkage of the cytoplasm seen in Fig. 7(a, e) is a result of morphological changes to the cells during chemical fixation. Chemical fixation causes the cells to lose matter and swell slightly, and as a result, the corresponding electron micrographs show gap formation by curdling and a decreased concentration of the cytoplasm (Kellenberger *et al.*, 1992). Binding of specific plasma proteins was determined by initially incubating the thin sections of bacteria with a primary antibody. These antibodies included anti-human fibrinogen, anti-kininogen AS88 (which probes for intact HK) and anti-NAT26 (which probes for a peptide sequence in domain D3 of HK). Binding of these antibodies was detected by incubation with a gold-labelled secondary antibody which could be visualized by electron microscopy (Fig. 7). Following exposure to plasma, cell morphology of *B. fragilis* and *B. thetaiotaomicron* was unaffected, as evidenced by an electron-dense cytoplasm (Fig. 7a, e). Immunostaining revealed that fibrinogen and HK are bound by both bacteria and, as judged from the images, the binding of these plasma proteins is surface-associated (Fig. 7b–d, f–h). Overall, these findings support the results demonstrating that both bacterial species caused activation of the contact system and that the mechanism involves HK binding. Also, both bacteria caused prolongation of the intrinsic pathway of coagulation, which could be a result of fibrinogen-binding and contact system activation. Hence, these images provide strong visual support for both HK and fibrinogen binding to the *Bacteroides* species tested.

## DISCUSSION

During the establishment of an infection, bacteria must first overcome many of the host's immune responses. One such response is activation of the contact system, which results in initiation of many procoagulative and proinflammatory cascades such as release of the proinflammatory peptide bradykinin and initiation of the intrinsic pathway of coagulation (Risberg *et al.*, 1991). Activation of the contact system also generates antibacterial peptides from domain D3 of HK (Frick *et al.*, 2006). However, over-activation of the contact system can have a deleterious effect on the host and be an important pathogenicity factor for invading pathogens. Large increases in vascular permeability enable invading pathogens to access more nutrients and colonize other tissue sites of the human host. Therefore, the ability of a pathogen to activate the contact system and thereby modulate the host immune response can increase virulence and confer advantages on the bacterium during infection.

In the present investigation, two species of the obligately anaerobic genus *Bacteroides*, *B. fragilis* and *B. thetaiotaomicron*, were found to interact with components of the contact system. Although normally a commensal organism, *Bacteroides* can cause significant opportunistic infections, including abscess formation and bacteraemia, with *B. fragilis* being regarded as the most virulent species (Wexler, 2007). Rapid binding and activation of the contact factors on the surface of *Bacteroides* resulted in cleavage of HK and release of bradykinin. In addition, activation of FXI at the bacterial surface initiated the intrinsic pathway of coagulation. When released during contact activation, bradykinin plays an important role in inflammation, and several studies suggest that an uncontrolled activation of the contact system contributes to the symptoms seen in sepsis (Bhoola *et al.*, 1992; Joseph & Kaplan, 2005). Several studies have reported efficient activation of contact factors by important pathogenic bacteria and/or their products. For instance, *Escherichia coli* and *Salmonella* species expressing curli fibres and M proteins of *Streptococcus pyogenes* are able to assemble contact factors on their surface, leading to bradykinin release (Ben Nasr *et al.*, 1995, 1996; Herwald *et al.*, 1998; Persson *et al.*, 2000). *Staphylococcus aureus*, a frequent causative agent of sepsis, can release bradykinin as a result of contact activation on the bacterial surface (Mattsson *et al.*, 2001) and also through secretion of cysteine proteinases that can cleave HK (Imamura *et al.*, 2005). Both *B. fragilis* and *B. thetaiotaomicron* caused significant levels of bradykinin to be released in human plasma, a mechanism that will provide the bacteria with an opportunity to spread during infection.

Fibrinogen is a major clotting protein of plasma, and recently a putative surface lipoprotein, BF-FBP, was identified as a fibrinogen binding protein in *B. fragilis* (Houston *et al.*, 2010). This protein displays very low homology (18 % identity) with the outer membrane 57.7 kDa hypothetical protein identified in this study as a putative fibrinogen binding protein, indicating that *Bacteroides* have evolved different proteins for adsorption of this key molecule of blood coagulation. Depletion of fibrinogen and contact factors in human plasma causes a hypocoagulative state (Ponjee *et al.*, 1991), and the significant delay in intrinsic, extrinsic and common pathway clotting times caused by both *Bacteroides* species tested in this study (see Fig. 5a–c) emphasizes that the bacteria adsorb these factors to its surface. In the case of *Salmonella*, an equivalent number of bacterial cells as used for *Bacteroides* caused a ~75 s delay in intrinsic clotting times and contact activation on its surface generated active thrombin (Persson *et al.*, 2000), which suggests that both *B. fragilis* and *B. thetaiotaomicron* are efficient inducers of contact activation. The observed prolongation of the tissue factor-driven coagulation further emphasizes that fibrinogen depletion by these *Bacteroides* species will prevent clot formation. In relation to the study carried out by Houston *et al.* (2010), it is interesting that a fibrinolytic activity was detected in *B. fragilis* culture supernatants. This is in contrast with the present investigation where no such activity was found (data not shown). However, different incubation times and enzyme concentrations may explain this discrepancy. Indeed, the observed prolonged thrombin-induced coagulation time following plasma exposure to *Bacteroides* might reflect not only depletion of fibrinogen but also proteolytic cleavage leading to inhibition of fibrin network formation.

The capsule in *B. fragilis* is regarded as playing a role in virulence and in abscess formation (Tzianabos *et al.*, 1993), and being involved in protection against phagocytic uptake and killing (Reid & Patrick, 1984). Interestingly, *B. fragilis* expresses multiple capsular polysaccharides and the bacterium is also able to modulate its expression of polysaccharides, a process regulated by phase variation (Krinos *et al.*, 2001). Furthermore, within an individual strain of *B. fragilis*, various encapsulated variants were observed. Recently, it was also shown that *Bacteroides* not commonly associated with opportunistic infection also contain diverse polysaccharide loci (Patrick *et al.*, 2010). Thus, the surface variation of capsular polysaccharides might have an impact on the binding of plasma proteins to outer-membrane-anchored bacterial proteins. This could help explain the low level of binding observed in some experiments.

Despite the high occurrence of *Bacteroides* in anaerobic infections and their levels of antibiotic resistance, little is known about their virulence mechanisms. The modulation of normal clot formation and bradykinin release described in this study represents a novel mechanism that may contribute to the pathogenicity of *Bacteroides*. Inhibition of clot formation under infectious conditions would allow the bacteria greater opportunities to enlarge the area of infection.
